# Forming Consensus To Advance Urobiome Research

**DOI:** 10.1128/mSystems.01371-20

**Published:** 2021-07-20

**Authors:** Linda Brubaker, Jean-Philippe F. Gourdine, Nazema Y. Siddiqui, Amanda Holland, Thomas Halverson, Roberto Limeria, David Pride, Lenore Ackerman, Catherine S. Forster, Kristin M. Jacobs, Krystal J. Thomas-White, Catherine Putonti, Qunfeng Dong, Michael Weinstein, Emily S. Lukacz, Lisa Karstens, Alan J. Wolfe

**Affiliations:** a Department of Obstetrics, Gynecology and Reproductive Sciences, Division of Female Pelvic Medicine and Reconstructive Surgery, University of California, San Diego, San Diego, California, USA; b Oregon Clinical & Translational Research Institute (OCTRI), Oregon Health & Science University, Portland, Oregon, USA; c Department of Obstetrics and Gynecology, Division of Female Pelvic Medicine and Reconstructive Surgery, Duke University, Durham, North Carolina, USA; d Department of Obstetrics and Gynecology, Oregon Health & Science University, Portland, Oregon, USA; e Department of Microbiology and Immunology, Stritch School of Medicine, Loyola University Chicago, Maywood, Illinois, USA; f Loyola Genomics Facility, Stritch School of Medicine, Loyola University Chicago, Maywood, Illinois, USA; g Department of Pathology, University of California, San Diego, San Diego, California, USA; h Department of Medicine, University of California, San Diego, San Diego, California, USA; i Department of Urology, Division of Female Pelvic Medicine and Reconstructive Surgery, University of California, Los Angeles, Los Angeles, California, USA; j Department of Pediatrics, University of Pittsburgh School of Medicine, Pittsburgh, Pennsylvania, USA; k Department of Obstetrics and Gynecology, Division of Female Pelvic Medicine and Reconstructive Surgery, Rush University, Chicago, Illinois, USA; l Department of Microbiology and Immunology, Stanford University School of Medicine, Stanford, California, USA; m Department of Biology, Bioinformatics Program, Loyola University Chicago, Chicago, Illinois, USA; n Department of Medicine, Stritch School of Medicine, Loyola University Chicago, Maywood, Illinois, USA; o Center for Biomedical Informatics, Loyola University Chicago, Chicago, Illinois, USA; p Molecular, Cell, and Developmental Biology, University of California, Los Angeles, Los Angeles, California, USA; q Zymo Research, Irvine, California, USA; r Department of Clinical Epidemiology and Medical Informatics, Oregon Health & Science University, Portland, Oregon, USA; Qingdao Institute of BioEnergy and Bioprocess Technology, Chinese Academy of Sciences

**Keywords:** consensus, guideline, human microbiome, research, statement, urinary microbiome, urobiome

## Abstract

Urobiome research has the potential to advance the understanding of a wide range of diseases, including lower urinary tract symptoms and kidney disease. Many scientific areas have benefited from early research method consensus to facilitate the greater, common good. This consensus document, developed by a group of expert investigators currently engaged in urobiome research (UROBIOME 2020 conference participants), aims to promote standardization and advances in this field by the adoption of common core research practices. We propose a standardized nomenclature as well as considerations for specimen collection, preservation, storage, and processing. Best practices for urobiome study design include our proposal for standard metadata elements as part of core metadata collection. Although it is impractical to follow fixed analytical procedures when analyzing urobiome data, we propose guidelines to document and report data originating from urobiome studies. We offer this first consensus document with every expectation of subsequent revision as our field progresses.

## OPINION/HYPOTHESIS

**FIG 1:**
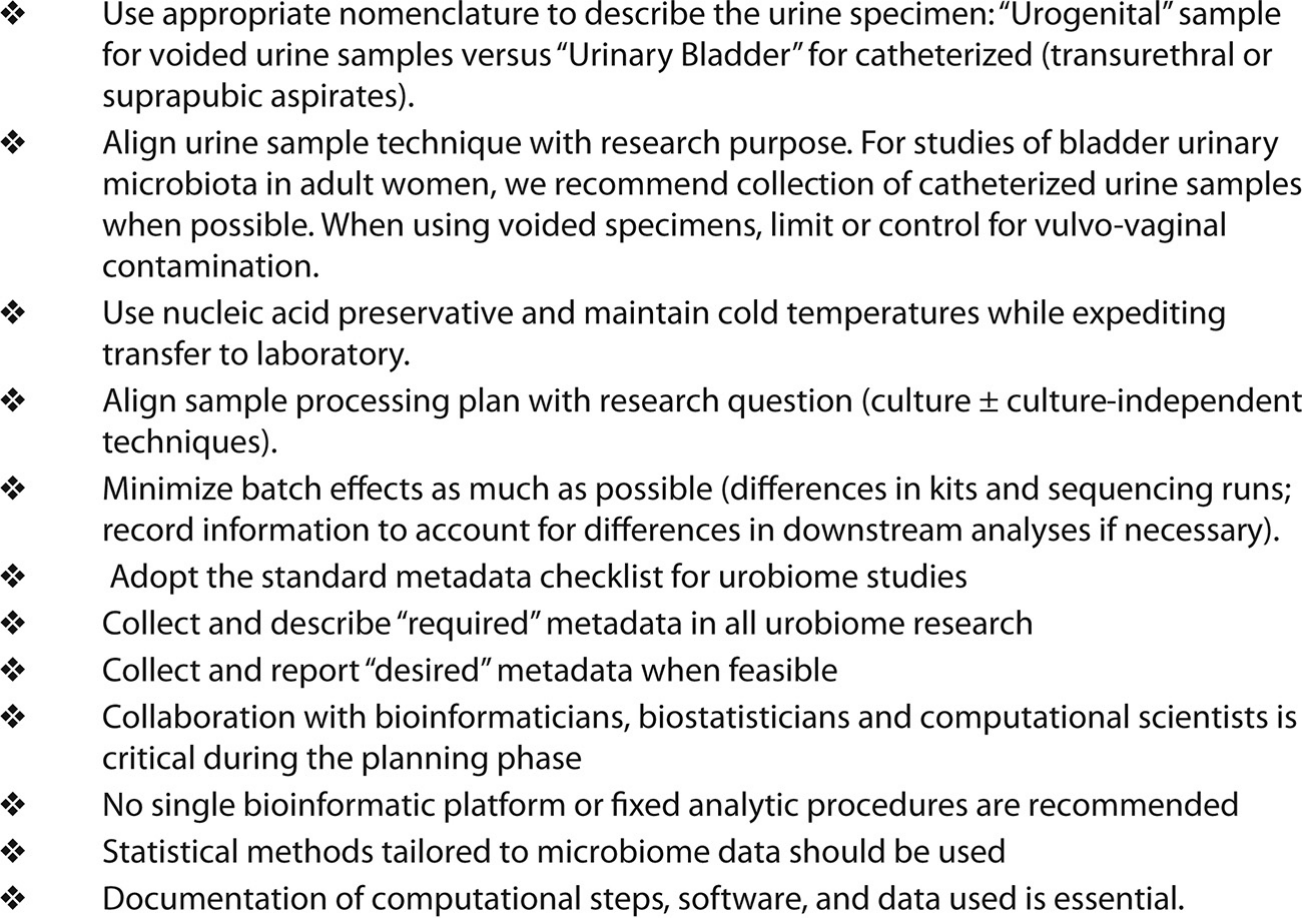
Key recommendations for urobiome research.

Since the discovery of the human urinary microbiome (urobiome), urobiome research has been impacted by inconsistent sampling conditions, technical conditions, and participant-related factors (1). The number of investigators currently working in urobiome research is still relatively small; however, the rapid growth of the field and the variety of approaches used to date have highlighted an urgent need for consensus on optimal strategies for the scientific investigation of the urobiome. A group of expert investigators currently engaged in urobiome research gathered to share research progress and exchange ideas at the National Institutes of Health (NIH)-sponsored international UROBIOME conferences in 2019 and 2020. This consensus document, developed by UROBIOME 2020 conference participants and their collaborators, aims to promote standardization and advances in this field by the adoption of common core research practices (Fig. 1).

## TERMINOLOGY

**FIG 2:**
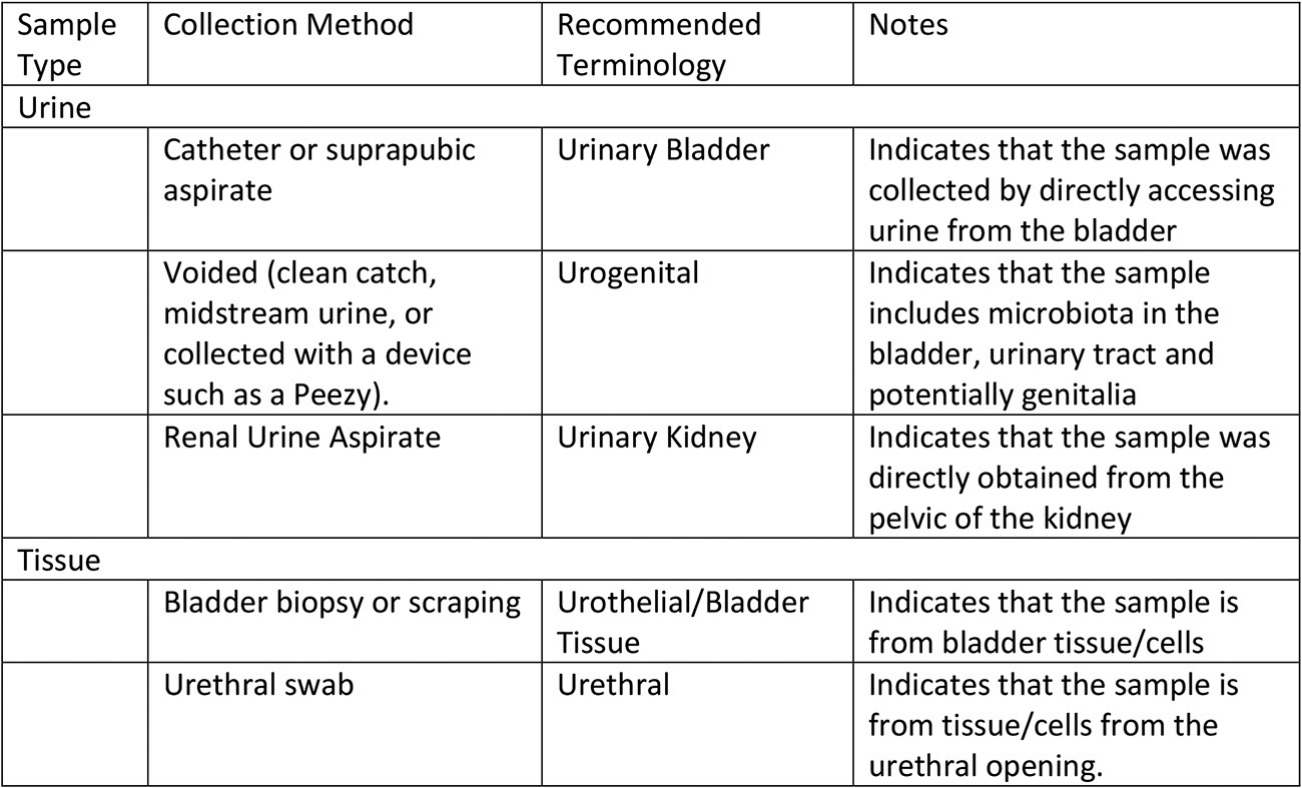
Recommended terminology for urobiome samples.

Standard terminology for urine specimens is necessary (Fig. 2). Many descriptors, including “bladder,” “urinary,” “urogenital,” and “genitourinary,” have been used, and these terms are often conflated. We propose a standardized nomenclature to explicitly describe the specimen as it relates to the collection method. The preferred, recommended terminology for a voided urine sample is “urogenital sample.” The preferred, recommended terminology for a catheterized urine sample (either transurethral or suprapubic) is “urinary bladder.” Samples obtained by urethral swabs, by urothelial/tissue biopsy, or from the kidney pelvis should be so named.

## URINE SPECIMEN COLLECTION

The urine specimen collection method must guide analysis and data interpretation, appropriately recognizing anatomical differences between sexes. Although the microbial biomass increases as the urine moves from the kidney to the bladder, urethra, and external genitalia, the urobiome has a low microbial biomass compared to other human microbial niches. Several studies have provided convincing evidence that the female urobiome includes vulvovaginal microbes (2, 3) when conventional “clean catch” midstream voided urine is used; thus, this type of sample should be referred to as a urogenital sample. A catheterized urine sample minimizes the inclusion of vulvovaginal microbes. When catheterization is not feasible or not desired (due to the potential disturbance of the urobiome itself) or when researchers wish to answer questions concerning the lower urinary tract microbiota, voided urine samples can be collected with a urinal device (i.e., Peezy midstream [Forte Medical]) that decreases microbial abundance and diversity, apparently by decreasing posturethral contamination (4). When multiple samples are collected from the same research participant, the order of collection should be specified. An alternative is to include a periurethral swab to allow the separate detection of genital microbes (5). Separate urethral swabs should be used for studies of the urethral microbiota. There is much less research informing urinary sample collection techniques in men; however, the currently available evidence supports the following conclusions: (i) the microbiome of voided urine most closely resembles that of urethral swabs, and (ii) catheterized urine does not tend to resemble voided urine (6, 7). Therefore, for males, we recommend using the term “urogenital” for voided urine and “urinary bladder” for catheterized urine or suprapubic aspirates.

## SPECIMEN PRESERVATION AND STORAGE

Immediately upon procurement, specimens should be cooled on ice or in a 4°C refrigerator and should be received by research personnel within 4 h. To avoid inappropriate microbial growth or degradation of nucleic acids, specimens should be appropriately preserved.

For all culture-based techniques, we recommend the use of BD Vacutainer Plus C&S boric acid sodium borate/formate (“gray top”) tubes (Becton, Dickinson and Company, Franklin Lakes, NJ). These are commonly used for clinical culture and antimicrobial sensitivity testing because they maintain microbial viability for at least 24 h under ambient conditions while inhibiting growth. This 24-h period gives research personnel some flexibility and permits overnight shipping.

For culture-independent analyses, we recommend the addition of AssayAssure (Sierra Molecular Corporation, Princeton, NJ) directly to the sample in a 1:10 ratio. This reagent is designed to inhibit 31 enzyme families known to degrade nucleic acids and thus stabilizes nucleic acids (DNA and RNA) over extended time periods without freezing or refrigeration. Importantly, it does not inhibit the amplification fundamental to PCR-based analyses such as 16S rRNA gene sequencing. It is recommended that the specimen be frozen at −80°C upon receipt. However, a benchmarking study showed that AssayAssure in combination with immediate cooling to 4°C or freezing at −20°C allowed storage for up to 4 days with a minimal impact on alpha diversity (8). Although the AssayAssure product guide states that samples can be maintained for up to 4 days at room temperature, we recommend caution when interpreting data from specimens held in this fashion compared to those immediately cooled in the presence of AssayAssure as different taxa may be recovered under different temperatures (8). We recommend rapid shipment (overnight if possible) on dry ice; however, the 4-day window allows flexibility as long as the samples remain cool. Other nucleic acid preservatives exist (e.g., DNA/RNA Shield [Zymo Research Corporation, Irvine, CA]) and can be used if AssayAssure is unavailable. As there is no current evidence that either pelleting/freezing bacteria or boric acid will adequately preserve nucleic acid, we recommend that this preservative/storage method should be included as a study limitation when nucleic acid preservatives are not utilized (due to affordability or other reasons). Studies reporting on urobiome findings should explicitly describe the use of preservative and storage conditions.

## SAMPLE PROCESSING

Traditional and enhanced culture techniques, as well as culture-independent methods, can be used for microbial detection. Culture techniques facilitate microbial detection and demonstrate that the microbe is alive, allowing subsequent experiments with the microbe itself. Lists of known urinary microbes and their growth conditions have been published (9, 10). Enhanced culture techniques, also known as metaculturomics, move beyond the traditional method described by Kass (11), allowing detection of microbes similar to that achieved with sequencing techniques (9, 10). Several enhanced culture methods have been reported (12, 13), including the expanded quantitative urine culture (EQUC) protocol, which has been used extensively for urobiome studies (9, 10, 13). To account for the very low biomass of catheterized urine specimens, we recommend plating 100 μl, which allows the detection of 10 CFU/ml. However, smaller volumes (1 or 10 μl) are recommended to achieve accurate counts of CFU per milliliter for voided urine samples or swabs (e.g., urethral or vaginal). Compared to the standard method, EQUC uses additional growth media (9, 10). The selection of media will depend on the research question, the cohort under study, the sample type, and resource constraints. The use of Columbia CNA (colistin naladixic acid) blood agar plates is critical to detect underlying Gram-positive bacteria that are often overwhelmed by more numerous and faster-growing Gram-negative bacteria such as Escherichia coli (9, 10). EQUC uses more atmospheric conditions than the standard method; 5% CO_2_ allows the growth of most urinary species, which prefer less oxygen. Anaerobic conditions are used for obligate anaerobes; when possible, we recommend an anaerobic chamber. If a chamber is not available, anaerobic jars can suffice for many but not all anaerobes. Finally, an extended incubation period (48 instead of 24 h) allows for the growth of slow-growing microbes and for the morphological differences between species to develop (8).

For sequencing, investigators should have a complete and detailed workflow (including nucleic acid isolation, library preparation, and sequencing) that aligns with the study hypotheses and bioinformatic analysis. Currently, marker gene (amplicon) sequencing is most commonly used for urobiome investigations. Studies of the bacterial communities rely on a hypervariable region of the 16S rRNA gene, while fungal community surveys target the internal transcribed spacer (ITS) region (14). Whereas amplicon sequencing can be used for taxonomic assignment and to determine relative quantities, shotgun metagenomic sequencing can provide insight into urobiome functionality and can also detect the viral fraction, which lacks a conserved marker gene (15).

Nucleic acid isolation techniques affect sequencing results, with some nucleic acid isolation kits showing biases that could specifically affect urobiome information (15). Enzymatic lysis is generally more reproducible among a range of laboratory environments (16). When establishing an enzymatic lysis protocol within a new laboratory, testing must be performed to determine whether the lysing enzymes contain nucleic acids from their manufacturing process (contaminants known as “the kitome”). Lysozyme and mutanolysin have been shown to contain a minimum amount of kitome contamination while having the best lysis efficiency (16).

Purification methodologies can be done with either silica column or magnetic bead protocols. Silica columns are easy to use; however, as they tend to shear DNA during extraction, they should be used only for short-read sequencing. Magnetic beads are easier to automate and can provide similar yields and purities (17, 18). We recommend that, whenever possible, all samples from entire projects be sequenced at once on the same machine to minimize technical variations. When that is not possible, we recommend that machines with the most similar chemistries/flow cells be used and that available reagent lot numbers be recorded so that these metadata can be considered during analysis. We recommend running positive-control samples with each batch to identify any differences due to the batch.

For 16S amplicon sequencing and Illumina’s paired-end 250-bp chemistry, one must choose between longer sequences that span multiple variable regions of the 16S rRNA gene (e.g., V1-V3) or shorter regions (e.g., V4). Longer regions possess more sequence information for downstream taxonomic assignment. However, sequence read quality diminishes at the ends. For shorter regions, this problem is reduced because the reads in both directions overlap, and sequencing errors can be eliminated by comparing complementary reads. For longer regions, poor-quality sequence overlap in the middle region can yield artifacts, which artificially increase sample diversity.

The choice of sequencing chemistries for whole-genome sequencing of purified isolates is important. Short-read chemistries (e.g., Illumina and Ion Torrent) are recommended if draft assemblies are sufficient. If complete genome assemblies are required, then long-read sequencing chemistries (e.g., PacBio or Nanopore) can be used to provide scaffolding to assemble data from the short-read chemistries (19).

## CORE METADATA AND UROBIOME STUDY DESIGN

In clinical research, standardized guidelines for reporting randomized trials and observational studies have led to increased reporting quality and transparency for readers (20–22). In microbiome research, metadata guidelines function in a similar capacity to improve transparency, enhance interpretation, and facilitate integration and comparison of results among studies (23–25). Readers should be able to understand the design, conduct, and analysis of a microbiome study in order to comprehend and interpret results. Detailed and thorough reporting of metadata, the information that describes a sampling event and subsequent data generation efforts, facilitates a shared understanding of the relevance of research findings. In addition, collection and reporting of a common, minimal set of metadata across different projects will foster data comparisons and analysis; they will facilitate comparisons across studies and combining of studies to allow more powerful meta-analyses.

Following a review of other consensus-based guidelines and based on iterative discussions within the urobiome research community, we propose standard metadata elements for urobiome studies. These include the minimum required metadata elements as well as those that are optional but highly desired for publication (Table 1). Since urobiome studies commonly involve human subject research, protected health information must not be included in the sequencing data or metadata.

**TABLE 1:** Proposed elements to be included in the minimum metadata standards for reporting of urobiome research

Element(s)	Required/desired	Description^*d*^
Biological elements		
Age	Required	Age in years or months/days if appropriate for infant/young child population^*b*^
Sex	Required	Biological sex; gender if relevant for the study
Antibiotic usage	Desired	There is a lack of knowledge about postantibiotic microbiome recovery; when possible, we recommend recording of use in the prior 3 months or length of time between last antibiotic exposure and sample collection
Hormone status	Desired	Pubertal stage^*a*^
		Pregnant/postpartum
		Menopausal status: perimenopausal, postmenopausal
		Also specify if taking supplemental hormones (estrogen) and route (oral, transdermal, or vaginal, etc.)
		Last menstrual period (if menstruating)
Contraception	Desired	Use of oral contraceptives, other hormonal or nonhormonal/barrier, or none
Body mass index	Desired	Body mass index at the time of the study visit, calculated from height and weight
Race, ethnicity	Desired	If possible, use standard terminology from sources such as the U.S. census and SNOMED CT
Surgery	Desired	Performed in the prior 3 months
		Prior GU surgeries
		Prior implanted GU materials
Birth details^*b*^	Desired	Gestational age
		Mode of delivery
		NICU stay
		Method of feeding
Medical history	Desired	Diabetes/prediabetes
		Other relevant medical comorbidities
		Use of steroids or immunosuppressant medications
		GU anatomical abnormalities
		Recurrent GU infections
		Recent GU instrumentation
Urine characteristics	Desired	pH, specific gravity, leukocyte esterase, blood

Environmental variables		
Method of collection	Required	Void, collection device (Peezy)
		Catheter (use of Mitrofanoff^*a*^)
		Suprapubic aspirate
Geographic location^*c*^	Required	Can be discrete, including geographic coordinates, or broad, such as region or country
Seasonal	Desired	Month of collection
Dietary	Desired	Consumption of a special diet, use of fiber supplementation, yogurt consumption
Sexual activity	Desired	Time interval between last sexual activity and sample collection, if sexually active

Technical variables		
Date and time of collection^*c*^ with conditions	Required	Used to ensure that samples stored at room temp for long periods are highlighted as such, potentially impacting the validity of results
		Ensure that the date is generic enough to be included or use a date range
Date and time of freezing	Required	Time interval between sample collection and freezing
		Omit if samples undergo immediate DNA extraction
Preservative	Required	If used, name
DNA extraction	Required	Method/kit used
Sequencing method^*c*^	Required	e.g., Illumina, Ion Torrent, Nanopore, PacBio, Sanger, pyrosequencing; include amplicon/variable region(s) used
Processing details	Desired	Including, but not limited to, details of sample transfer method and extraction protocol (sterile hood or technique), etc.

a Additional recommendation for pediatric populations.

b Additional recommendation for infant populations.

c Required when uploading sequence data to the Sequence Read Archive (SRA) (27) or the European Nucleotide Archive (ENA) (36) public data repository.

d OCP, oral contraceptive pill; GU, genitourinary; NICU, neonatal intensive care unit.

Within the proposed metadata elements, “required” elements refer to the absolute minimum information needed to make data interpretable. The “desired” elements include characteristics that enhance the reader’s ability to interpret findings within specific cohorts. These elements have been associated with differences in microbiota in previous studies and thus are considered potentially confounding elements. We suggest that study teams aiming for a high level of rigor should collect information pertaining to the desired elements and either include this information when disseminating their research or explain the lack of inclusion. Researchers are highly encouraged to consider additional items relevant to their study design or specific research question. The recommended metadata elements in Table 1 are organized based on important biological, environmental, and technical factors that could introduce variability or confound results.

For studies that include marker gene sequencing (e.g., 16S rRNA gene sequencing), we have complied with the Genome Standards Consortium (GSC) recommendations for minimum information standards (MixS) for describing and publicly sharing these data (26). In collaborating with the GSC, we have created an environmental package (MixS-Urobiome) consisting of a checklist for describing minimum and desired information about marker gene analyses (26). Table S1 in the supplemental material displays a checklist structured to facilitate the uploading of information to public databases such as the Sequence Read Archive (SRA), where raw sequencing data are often shared (27). A Research Electronic Data Capture (REDCap) database template encompasses required and desired metadata elements should study teams wish to use a standard template for prospective studies (28).

10.1128/mSystems.01371-20.1 TABLE S1 Checklist to facilitate the uploading of information to public databases. Download Table S1, XLSX file, 0.04 MB. Copyright © 2021 Brubaker et al. 2021 Brubaker et al. https://creativecommons.org/licenses/by/4.0/ This content is distributed under the terms of the Creative Commons Attribution 4.0 International license.

## BIOINFORMATIC APPROACHES AND DATA ANALYSIS

Analyzing urobiome data is often tailored to the specific research questions addressed in a particular project, making it impractical to follow fixed analytical procedures. Table 2 displays guidance for documenting and reporting urobiome study data (29, 30). To ensure that urobiome data are appropriately handled and interpreted, it is essential to collaborate with bioinformaticians or computational biologists; consultation in the early stages of study design is recommended.

**TABLE 2:** Guidelines for processing sequencing data for urobiome research^*a*^

Data processing step	Description (reference[s])
Marker gene sequencing	
Grouping reads	Sequencing reads can be grouped into OTUs or ASVs; ASVs offer several advantages over OTUs, such as better accuracy and resolution, and hence are preferred (37); current ASV algorithms include DADA2 (38) and Deblur (39); significantly outdated OTU clustering algorithms (such as uclust [40]) should be avoided
Assigning taxonomy	Algorithm: taxonomy can be assigned with taxonomic classifiers such as naive Bayes or BLCA classifiers (41, 42); species-level assignment needs to be performed with algorithms designed for species-level assignments, such as BLCA or the exact matching approach implemented in DADA2 (43)
	Database: the Silva (42) and NCBI 16S (44) databases are preferred, as they are more representative of microbiota in the urobiome than the currently available version of the Greengenes database (v13_8) (43)
Data cleaning	Chimeras: chimeras arise from PCR and should be removed using an algorithm such as ChimeraSlayer (45) or UCHIME (37, 46)
	Contaminants: since catheter-collected specimens are typically low-biomass specimens, computational strategies for bacterial contaminants, identification, and removal should be used; Decontam is currently the preferred approach in conjunction with an exptl design that includes negative controls and/or a mock microbial dilution series to evaluate performance (47)

Whole-genome sequencing	
Data cleaning	Host DNA needs to be removed using tools such as Bowtie2 with the current human reference genome (48)
Read processing	Sequencing reads can be processed using metagenomic *de novo* sequence assembly using tools such as metaSPAdes (49) or binned, where reads are clustered by sequence similarity, using tools such as MaxBin (50)
Annotation	Taxonomic annotation: marker genes such as 16S rRNA and well-characterized functional genes can be used for genus- and species-level annotations using tools such as Metaphlan (51)
	Gene annotation: identifying relevant features of bacterial genomes can be performed using tools such as Prokka (52)
	Metabolic pathway analysis: the metabolic functional potential of a microbial community can be modeled and explored using tools such as CarveMe (53); as with marker gene sequencing, annotation is highly dependent on the reference databases used and how well the urobiome microbiota are represented

Software pipelines for data analysis	
Marker genes	QIIME2 (54), mothur (55), and DADA2 (38)
WGS	MG-RAST (56), EBI MetaGenomics (57), and IMG/M (58)
Viral	Classification of eukaryotic viruses and bacteriophage: Virmine (59)
	Classification of bacteriophage: VirSorter (60)

a OTUs, operational taxonomic units; ASVs, amplicon sequence variants; WGS, whole genome sequencing.

Several manipulations are needed to distill sequencing reads into biologically meaningful data for statistical analysis. Standard steps include quality filtering and denoising, grouping sequences by similarity for marker gene studies or binning approaches for whole-genome sequencing (WGS) studies, assembly for WGS studies, removing technical artifacts and noise, and assigning taxonomy (31). While the approach for a specific study depends on the data generated, the steps can be completed using freely available sequence processing platforms. Table 2 displays current guidelines and recommendations.

Urobiome studies are typically limited by a small sample size yet a large number of measured variables (taxa or genes). Thus, ecological community analyses such as alpha diversity (e.g., the Chao1, Simpson, Shannon, and Pielou indices) and beta diversity (e.g., Bray-Curtis and UniFrac) using nonmetric multidimensional scaling (NMDS) and principal-coordinate analysis (PCoA) are applied for multivariate analyses of microbiomes (32). These measures can identify overall differences between study groups. Drilling down to the level of taxa or genes is often desired, but the process is complex. Although standard statistical methods are often applied, it is important to realize that these methods are often not suitable because urobiome data are compositional, multivariate, nonnormal, highly skewed, and zero inflated. Therefore, we encourage the use of statistical methods tailored to microbiome data (33). Multiple-test correction is important for controlling for false positives in statistical analyses; however, these efforts may diminish real scientific findings. Thus, we recommend that investigators report raw and corrected *P* values and provide scientific justification for results that should be subject to further investigation and validation. Furthermore, it is important to realize that the exploratory nature of most urobiome projects (at least at the initial phase) makes defining a meaningful “effect size” *a priori* required for sample size calculation challenging.

To ensure the reproducibility of an analysis, documentation of computational steps, software, and data used is essential (34). For example, analysis performed in the R statistical programming language can be documented in RMarkdown (35). This documentation can be shared as supplemental material or stored on a code repository such as GitHub. Both raw data and the associated metadata should be deposited in public repositories for reanalysis (26). In the manuscript methods, software details should be appropriately mentioned and referenced (Table 3).

**TABLE 3:** Minimum information for reporting bioinformatics methods in urobiome studies

Information to be included	Description (reference)^*a*^
Software	Include software package and version; if using a package such as QIIME (61), reference key algorithms for OTU/ASV generation, taxonomy assignment, chimera removal, and contaminant detection
Databases	Include databases used and version
Code	Include essential custom-written code for analysis or data processing as supplemental material or link to code repository such as GitHub
Data	Raw sequencing data: stored in a public repository such as SRA (27), ENA (36), or dbGaP (62)
	WGS assemblies: stored in a public repository such as GenBank
	Metadata: follow MIMARKS (26) or MixS guidelines; upload with raw data

a SRA, Sequence Read Archive; ENA, European Nucleotide Archive; dbGaP, Database of Genotypes and Phenotypes.

## CONCLUDING COMMENTS

Urobiome research has the potential to advance our understanding of human health and a wide range of diseases, including lower urinary tract symptoms and kidney disease. Many scientific areas have benefited from early consensus on research methods by allowing investigators to more appropriately compare their findings with those of their colleagues, optimizing transparency and communication and facilitating research for the greater, common good. We offer this first consensus document with every expectation of subsequent revision as our field progresses.
